# Complete chloroplast genome of *Sorbaria sorbifolia* var. *stellipila*

**DOI:** 10.1080/23802359.2019.1642156

**Published:** 2019-07-16

**Authors:** Sang-Chul Kim, Ji-Young Ahn, Jei-Wan Lee

**Affiliations:** Department of Forest Bio Resources, National Institute of Forest Science, Suwon-si, Republic of Korea

**Keywords:** *Sorbaria sorbifolia* var. *stellipila*, Rosaceae, complete chloroplast genome, phylogenetic analysis

## Abstract

The complete plastid genome of *Sorbaria sorbifolia* var. *stellipila* was sequenced and analyzed in this study. It was found to be 160,820 bp and consisted of a large (88,869 bp) and small (20,853 bp) single-copy regions, separated by a pair of identical inverted repeats (25,549 bp). The GC content of the whole genome was 37.8%, and there were 84 unique protein-coding genes, 37 tRNAs, and 8 rRNAs. The gene order and organization were consistent with those of other complete plastid genomes from the Rosaceae. The phylogenetic tree construct was based on 76 protein-coding genes and demonstrated a sister relationship with the Rosaceae.

There are seven species in the genus *Sorbaria* (Ser.) A. Braun, a member of the Rosaceae group (https://www.theplantlist.org [cited 2019 Jun 20]. *Sorbaria sorbifolia* var. *stellipila* Maxim. is a deciduous shrub that grows naturally in the mountain valleys and wetlands of northeastern East Asia and reaches 2 m in height (Lee 1989). It is reported to be used medicinally to relieve the pain from fractures and bruises (Ahn 2000). In addition, the fruit of *S. sorbifolia* var. *stellipila* are known to have antioxidant activities that are three times greater than those of vitamin E owing to the complex actions of their active antioxidant ingredients (Park et al. 2011). However, molecular studies of this species have not yet been conducted. In this study, we have reported for the first time, the entire sequence of the chloroplast genome of *S. sorbifolia* var. *stellipila* and constructed its phylogenic tree.

Fresh leaves were collected from Hongneung Arboretum (37°35′N, 127°2′E) and genomic DNA was isolated from fresh leaves by using a Plasmid SV mini kit (GeneAll, Seoul, Korea). Extracted DNA was stored in the Plant DNA Bank of the National Institute of Forest Science (No. 0335132685; Suwon, Korea). Whole genome sequencing was conducted using the Ion Torrent sequencing platform (Life Technologies, Carlsbad, CA). Filtered sequences were assembled using *Prunus yedoensis* as the reference sequence (GenBank accession number KU985054). The sequenced fragments were assembled using Geneious R10 (Biomatters Ltd, Auckland, New Zealand; Kearse et al. 2012). Annotation was performed using DOGMA (http://dogma.ccbb.utexas.edu/) and BLAST searches. All of the tRNA sequences were confirmed using the web-based tool tRNAScan-SE (Schattner et al. 2005) with the default settings to corroborate the tRNA boundaries identified using Geneious. The complete chloroplast genome of *S. sorbifolia* var. *stellipila* was submitted to the NCBI database under the accession number MN026875.

The plastid of *S. sorbifolia* var. *stellipila* was found to have a double-stranded, circular DNA, with 160,820 bp, and contained two inverted repeat regions (IRs) of 25,549 bp, each of which was separated by large single-copy (LSC) and small single-copy (SSC) regions of 88,869 and 20,853 bp, respectively. The genome contained 129 genes, including 84 protein-coding genes, 37 tRNA genes, and 8 rRNA genes. Six of the protein-coding genes, 8 tRNA genes, and 4 rRNA genes were duplicated in the IR regions. The overall GC content was 37.1% (LSC, 33.7%; SSC, 29.7%; IRs, 42.9%).

The maximum-likelihood (ML) tree searches and ML bootstrap searches were performed using the RAxML BlackBox web-server (https://raxml-ng.vital-it.ch/#/, Stamatakis et al. 2008) with 76 protein-coding genes, and 16 of which those were identified to be from the Rosaceae (with the outgroup being Rhamnaceae). The RAxML analyses were run with rapid bootstrap analysis using a random starting tree and 100 ML bootstrap replicates. The genus *Sorbaria* was found to be a sister to *Chamaebatiaria millefolium* (Torr.) Maxim. (Figure 1).

**Figure 1.**
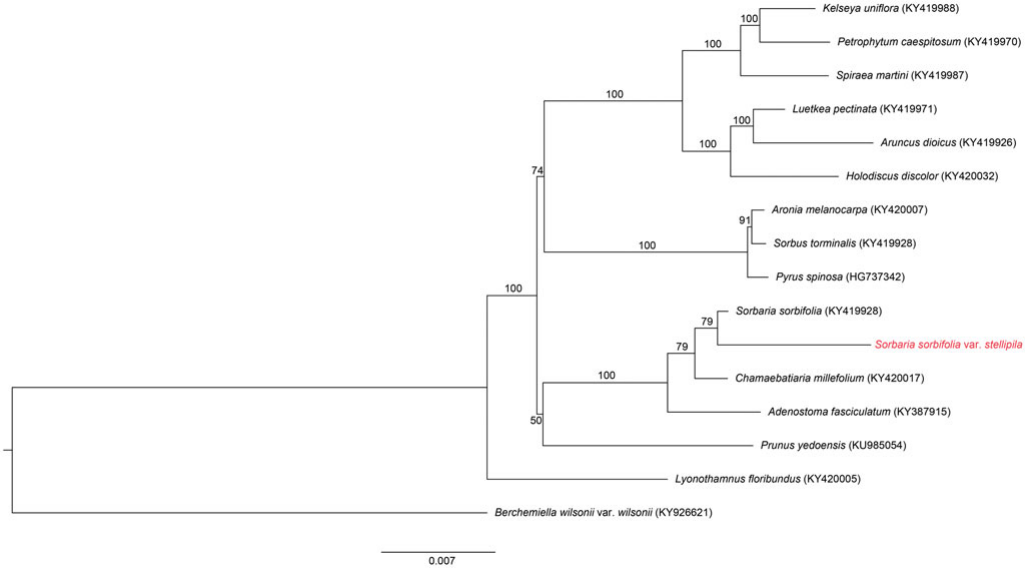
The maximum-likelihood (ML) tree based on the 16 representative chloroplast genomes of Rosaceae (with Rhamnaceae as the outgroup). The bootstrap value based on 100 replicates is shown on each node.
